# Flood risk management in Rwanda: A review of basic conditions and recommendations for an enhanced institutional framework

**DOI:** 10.4102/jamba.v18i1.2102

**Published:** 2026-10-15

**Authors:** Leonard Nzabonantuma, Erik Nilsson, Umaru G. Wali, Magnus Larson

**Affiliations:** 1 Department of Building and Environmental Technology, Faculty of Engineering, Lund University, Lund, Sweden; 2 Department of Civil, Environmental, and Geomatics Engineering, School of Engineering, College of Science and Technology, The University of Rwanda, Kigali, Rwanda; 3 United Nations University Hub on Water in a Changing Environment (WICE), United Nations University Institute for Water, Environment and Health, Faculty of Engineering, Lund University, Lund, Sweden

**Keywords:** flood risk management, catchment-based management, nature-based solutions, institutional framework, climate resilience, Rwanda

## Abstract

Rwanda faces substantial and growing challenges from flood impacts in both rural and urban areas. This review examines riverine flood events and flood risk management (FRM) in Rwanda, identifying key drivers, institutional developments and persistent gaps. Frequent flooding, which has affected more than 2 million people, is primarily driven by steep topography and intense rainfall, compounded by rapid urbanisation, deforestation and unregulated mining activities. Based on a systematic review of 19 stakeholder reports and 41 peer-reviewed research articles, the study highlights critical shortcomings in current FRM practices, including fragmented data management, the absence of dedicated FRM legislation and limited technical capacity at sub-national levels. Although existing interventions increasingly incorporate nature-based solutions such as terracing and reforestation, their effectiveness is often constrained by governance structures that follow administrative rather than hydrological boundaries. To support Rwanda’s Vision 2050, the review advocates a shift from reactive disaster response towards proactive, catchment-based flood governance.

**Contribution:**

Key recommendations include the establishment of technically specialised catchment-level institutions, the deployment of automated telemetry-enabled hydro-meteorological monitoring systems and the expansion of integrated green-grey infrastructure. Collectively, these measures aim to establish a robust, multi-level FRM framework capable of reducing vulnerability under escalating climate risks.

## Introduction

Over the past decade, flood events in Rwanda have caused severe impacts occurring almost yearly during the rainy season (Mind’je et al. 2019). During this period, floods and droughts have increased, affecting more than 2 million people (Rwanda Environment Management Authority [REMA] 2019). Ministry in Charge of Emergency Management (MINEMA) reports from 2016 to 2023 indicate that flood events across Rwanda were associated with significant losses (Iradukunda et al. 2025a), with 259 recorded deaths and 4338 damaged houses. Heavy rains are among the leading causes of excessive flooding that spills out of natural or artificial confines, causing erosion and landslides through intensive runoff (Mind, Li & Nsengiyumva 2020). The intricate patterns and spatial distribution of rainfall are influenced by several key factors, primarily the El-Nino-Southern Oscillation (ENSO), the Indian Ocean Dipole (IOD) and the underlying topography. These climatic phenomena play pivotal roles in shaping precipitation dynamics across regions, affecting both the timing and intensity of rainfall events (Abimbola et al. 2017; Jonah et al. 2021). The average annual rainfall of about 1200 mm for the entire country ranges from 800 mm in the East to 2000 mm in the high-altitude areas of the North and West (Ndekezi 2012).

In addition, population growth is straining land and degrading soils as well as the environment (De la Paix et al. 2013). Rwanda’s population has multiplied over the last seven decades, from 1 595 400 in 1934 to 13 200 000 according to the fifth population and housing census conducted in 2022 (National Institute of Statistics of Rwanda [NISR] 2023). Moreover, from 2012 to 2022, the population increased with a growth rate of 2.3%, and, if the current population growth rate continues, the population will reach 23 200 000 inhabitants and a population density of 881 people per km^2^ by 2050 (NISR 2023). This increase in population has led to a demand for land, converting natural forests into agricultural land (Kayitesi, Guzha & Mariethoz 2022) and plantations and for firewood (De la Paix et al. 2013). Statistics from the Forest Department reveal that the forest coverage was estimated to 658 500 ha in 1996 and reduced to 240 746 ha by 2007; a loss of 64% was observed between 1960 and 2007, which is more than 1.3% per year (Habiyaremye et al. 2011).

Flood hazards are also exacerbated by unplanned urbanisation, land use (Mind’je et al. 2019) and geomorphological processes, as streams and riverbeds undergo aggradation due to deposited sediment, which reduces water depth and leads to flooding during heavy rainfall (Nones 2019) and alters water quality (Kulimushi et al. 2021). Most of the sediments deposited in the rivers originate from mining activities and intensive farming on steep slopes (Kayitesi et al. 2022).

Previous studies have also concluded that community participation and ownership is a concern in flood risk management (FRM) in Rwanda. The sense of collective responsibility for flood risk reduction is weaker in rural than in urban areas, which may result in an increased flood risk in those areas (Mind’je et al. 2019). Another key limitation for an effective FRM is data availability. The limited availability of reliable climate data, combined with the country’s complex topography and localised climatic variations, makes Rwanda particularly challenging to represent accurately in climate models (Abimbola et al. 2017).

This article examines the evolution of FRM in Rwanda from 2010 to 2025, focusing on the key drivers of flooding and the strategies employed to mitigate these risks. Flood drivers are categorised into environmental and anthropogenic factors. Additionally, the paper evaluates the roles of key stakeholders in shaping FRM policies and practices. It examines decision-making processes across institutional levels, from local district authorities to national and international bodies. Based on the findings, the paper offers actionable recommendations to enhance institutional frameworks and strengthen FRM in Rwanda.

## Research methods and designs

### Overview

The methodology was designed to provide a systematic and comprehensive analysis of the key factors influencing flood risks and the corresponding management strategies. As outlined in the flowchart (Figure 1), the approach followed a multi-step process that integrated data from sources from both research and stakeholder publications, ensuring a thorough evaluation and synthesis of relevant information. The included publications were reviewed to synthesise information on flood risk related to the thematic areas presented in Figure 1: socio-economic impacts, environmental and anthropogenic flood factors, institutional framework, implemented measures and data availability. From the findings in the thematic areas, key issues and challenges were identified followed by recommendations for improved institutional framework, mitigation measures, data and monitoring and research.

**FIGURE 1:**
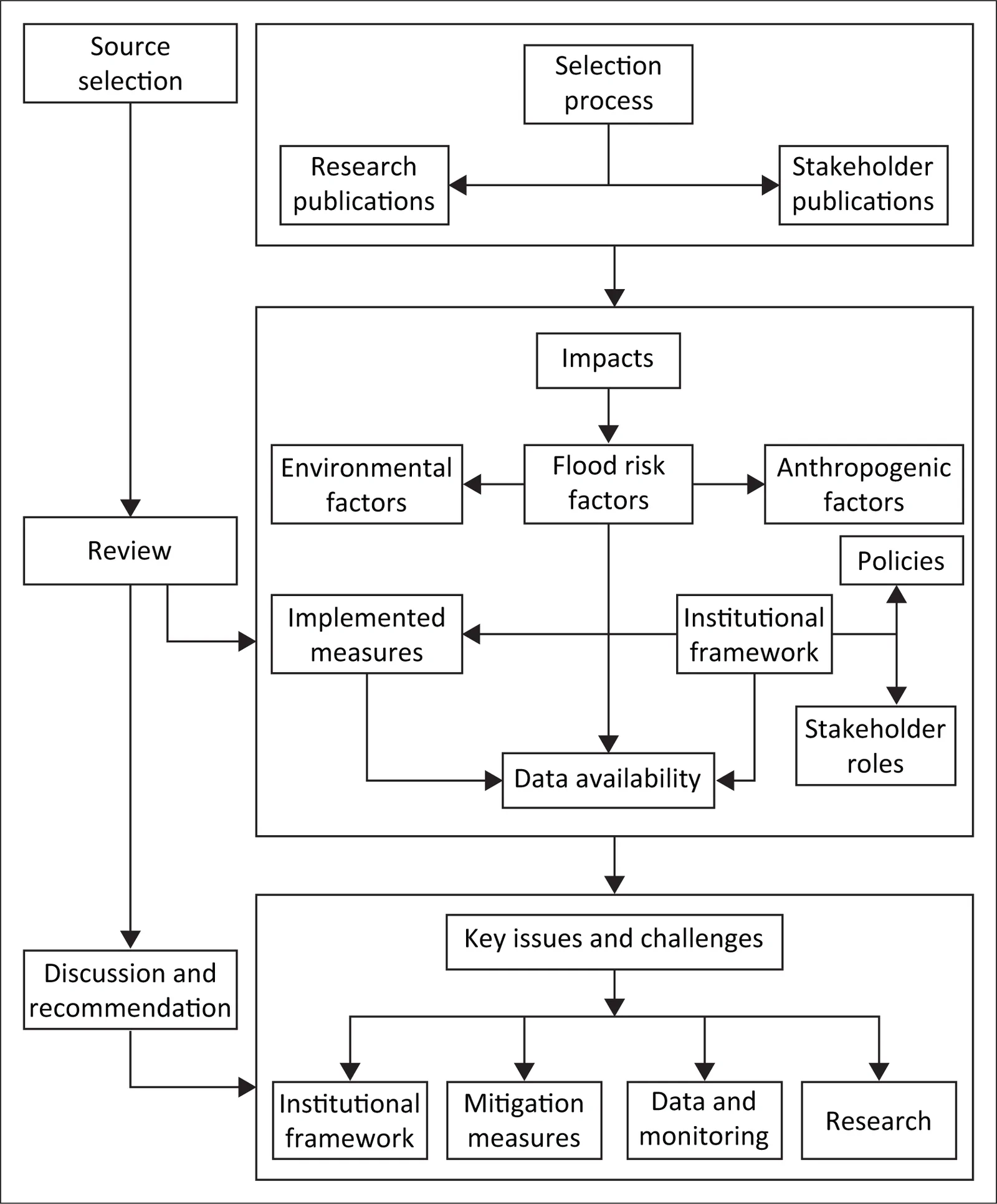
Flowchart of the methodology.

The analysis was conducted to generate results that can support Rwanda’s national strategies and the United Nations’ Sustainable Development Goals (SDGs), with a particular focus on the national target of eradicating extreme poverty and hunger by 2030.

### Stakeholder publications

The stakeholders covered in this review are public institutions, companies, non-governmental organisations and local communities involved in disaster management. Stakeholders were identified by combining lists published by Ministry of Natural Resources (MINIRENA) and MINEMA (MINEMA 2023b). The merged list contained 16 stakeholders, mainly public institutions (Appendix 1). For each stakeholder, their websites were visited, and all publications from 2010 onward covering flood-related strategies or potential causes of floods were reviewed. The year 2010 was set as the starting point for the review, considering that it marked the establishment and initial reporting of most water management institutions in Rwanda, coinciding with the onset of their activities focused on monitoring environmental degradation and disaster management. With the above criteria, 70 publications were identified and were included for detailed review. These publications consisted of 16 reports, two statutes, one strategic plan and others. Most of the reports outline the primary drivers of the environmental changes, assess the status and trends in environmental conditions (floods, water pollution and degradation of the other ecosystems) in Rwanda and formulate recommendations for sustainability of implementation of mitigation measures and approaches. Additionally, catchment management plans present strategies for the rehabilitation of the catchments and for sustaining ecosystem services. Statutes aimed at creating laws and policies were also scrutinised. Identified gaps are detailed in the findings and recommendations section, whereas Table 2-A1 (see Appendix 1) lists all reviewed publications and summarises their findings.

### Research publications

A comprehensive literature review was conducted to examine FRM in Rwanda. The search encompassed Web of Science, ScienceDirect, Springer, Wiley, Taylor & Francis and MDPI databases, focusing on peer-reviewed articles published between 2010 and 2025. The keywords used were ‘flood hazard’, ‘flood damages’, ‘flood risk management’ and ‘Rwanda’. The initial search yielded 90 articles, which were further refined to 28 articles specifically focusing on floods in Rwanda. To provide and support the development of FRM recommendations, an additional 13 peer-reviewed papers on FRM from sub-Saharan Africa and other regions with similar social and environmental conditions were included.

### Ethical considerations

This article does not contain any studies involving human participants and/or animals performed by any of the authors.

## Results

### Impacts

The impact of floods in Rwanda has been considerable, with evidence indicating that such events have led to severe environmental degradation, socio-economic disruptions and slowed economic growth (Habumugisha & Mukashyaka 2023). Figure 2 presents the annual crop damages caused by floods at the district level between 2016 and 2024, based on data from MINEMA (2024a). Among the different indicators of flood severity, crop damage serves as a particularly informative measure, as it reflects both the spatial extent of inundated areas and the frequency of flood impacts across districts. The data reveal that flood-related crop losses occur every year, affecting all districts nationwide, with notably severe impacts recorded in 2018. Districts situated in the Northern, Western, Central and Eastern regions appear to be disproportionately susceptible to flood events compared to other parts of the country (Mind’je et al. 2019; MINEMA 2024a).

**FIGURE 2:**
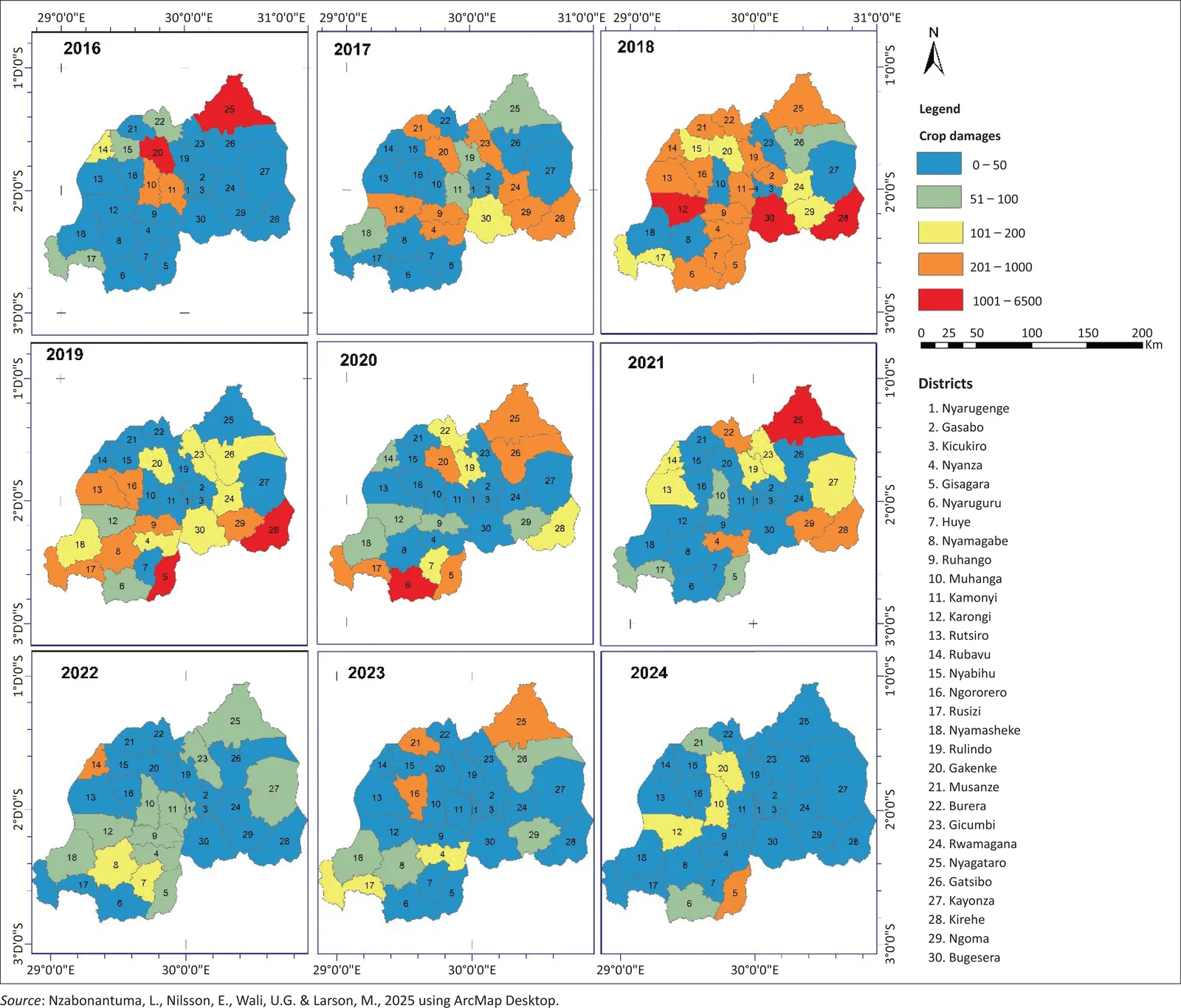
Annual crop damages by flood (including also crop damages by hailstorm and rainstorms) in Rwanda from 2016 to 2024.

In addition to impact reports, MINEMA has published annual reports on disaster occurrences in the country since the beginning of 2016. The report Rwanda Rapid Post Disaster Needs Assessment (Government of Rwanda (GoR) 2019) revealed that for the period 2016–2019, floods and landslides were the most devastating natural hazards, with the peak in terms of geographical coverage, severity of damage and extent of loss recorded in the first 5 months of 2018. While 15 districts were directly affected in this period, the whole country suffered from the effects. A considerable number of people died, and others were seriously injured, while many families became homeless. Damage to roads and bridges disrupted business, and in 2018 alone, the total impact of floods and landslides was estimated at USD 256 600. Flood disasters have had significant economic ramifications, impacting various sectors and the country’s overall economic growth. It is reported that over the past two decades, an estimated 2 730 554 people were affected, 31 580 temporarily displaced, 221 killed, and 252 seriously injured, resulting in significant economic losses (Government of Rwanda (GoR) 2019).

Figure 3a, b and d–f illustrate examples of some affected areas across the country during the destructive flood event on 02–03 May 2023, primarily in the North-Western region. On the night of 03 May 2023, a record-breaking rainfall intensity of 182.6 mm was recorded at Mushubati rain gauge station (in Rutsiro District), and the ensuing flood disaster across the country resulted in 135 deaths, 5963 damaged houses and 51 497 displaced people (Idukunda et al. 2025a; MINEMA 2023a). These flood events, accompanied by landslides, caused extensive damage across Rwanda’s Western, Northern and Southern Provinces. Figure 3a shows the Satinstyi River bridge, which became clogged with sediments and lost its functionality. Figure 3b, d, and e depict the destruction of an asphalt road and damage to houses, respectively, in Nyabihu and Rubavu Districts. The total damage and losses estimated at 215.8 billion Rwandan francs (equivalent to USD 187 million), with recovery needs projected at 629.38 billion Rwandan francs (equivalent to USD 547.29 million). Direct production losses amounted to approximately 36.1 billion Rwandan francs (equivalent to USD 31.1 million) (International Monetary Fund 2023). The International Monetary Fund estimated that the floods contributed to a decline in Rwanda’s real gross domestic product (GDP) growth from 6.2% to 5.8% in 2023 (International Monetary Fund 2023). Another recent flood event occurred in May 2020 in Kigali City and the Southern Province, with examples of flood conditions shown in Figure 3c for a bridge and road connecting Kigali City to the Southern province, where traffic was cutoff on the night of 07 May 2020. Additionally, recent estimates suggest that climate change could reduce Rwanda’s GDP by 5%–7% below the baseline by 2050, with severe flooding potentially amplifying this reduction by an additional 4.4% (The World Bank 2024).

**FIGURE 3:**
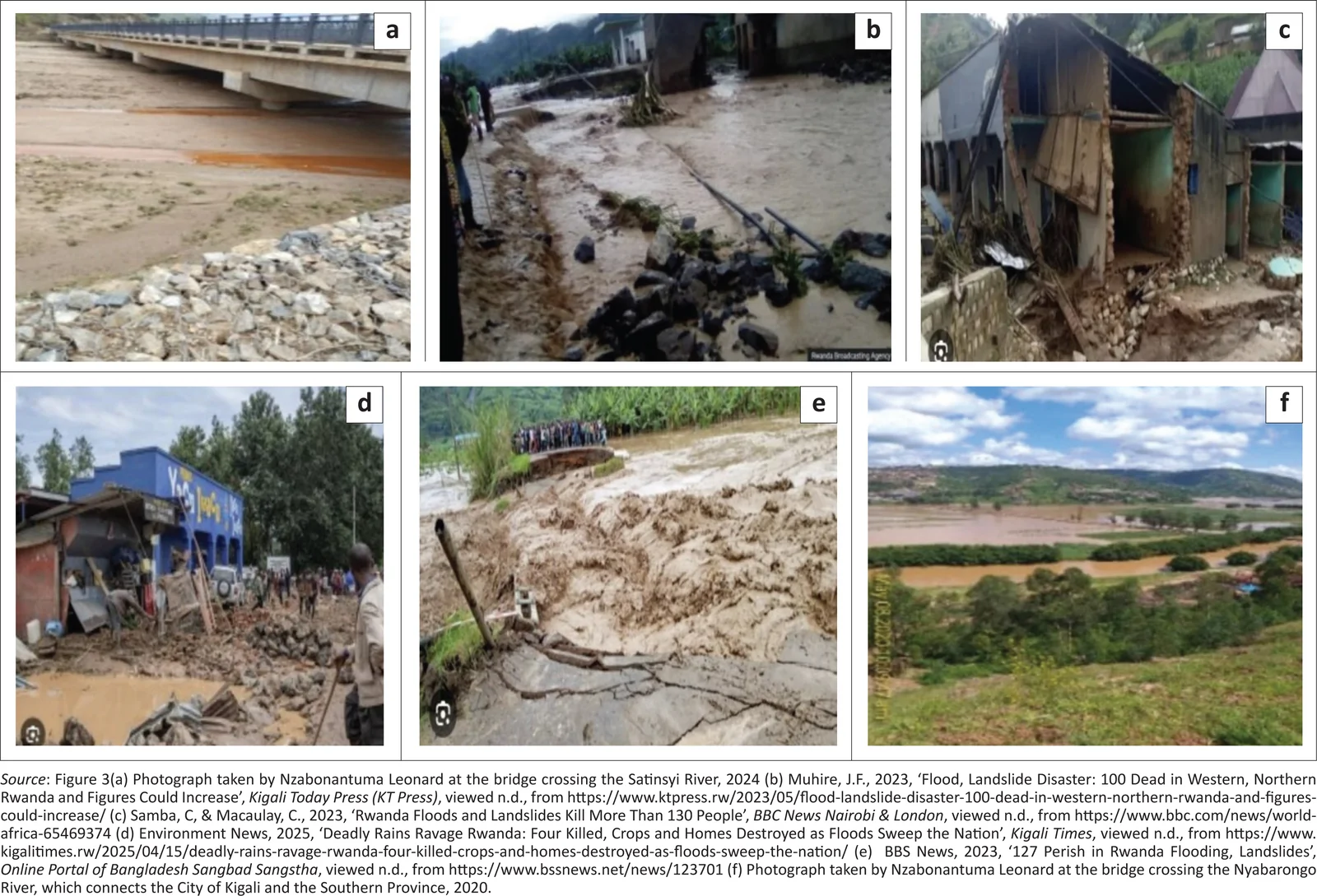
Examples of flood-affected locations by the flood events in May 2020 and May 2023: (a) bridge at Satinstyi river full of sediments and alluvium in May 2023, (b, c, d) destroyed asphaltic road and damaged houses in Rubavu district in May 2023, (e) damaged asphaltic road in Nyabihu district by the flood event of May 2023, and (f) overwhelmed bridge and road joining Kigali City and South province where there was a traffic cutoff in May 2020.

### Flood risk factors

#### Environmental factors

Rwanda’s topography, characterised by steep hills, plays a pivotal role in shaping its weather patterns. Figure 4 illustrates how elevation is linked to monthly rainfall distribution across the country. In the high mountains of the North-West and South-West regions, rainfall is plentiful, gradually diminishing towards the South-East (Jonah et al. 2021; Uwizewe et al. 2024). These topographic and hydrological features, such as slope steepness and elevation gradients, are key factors contributing to the heightened risk of flooding, as they determine how water moves and accumulates during periods of precipitation (Ntwali, Ogwang & Ongoma 2016).

**FIGURE 4:**
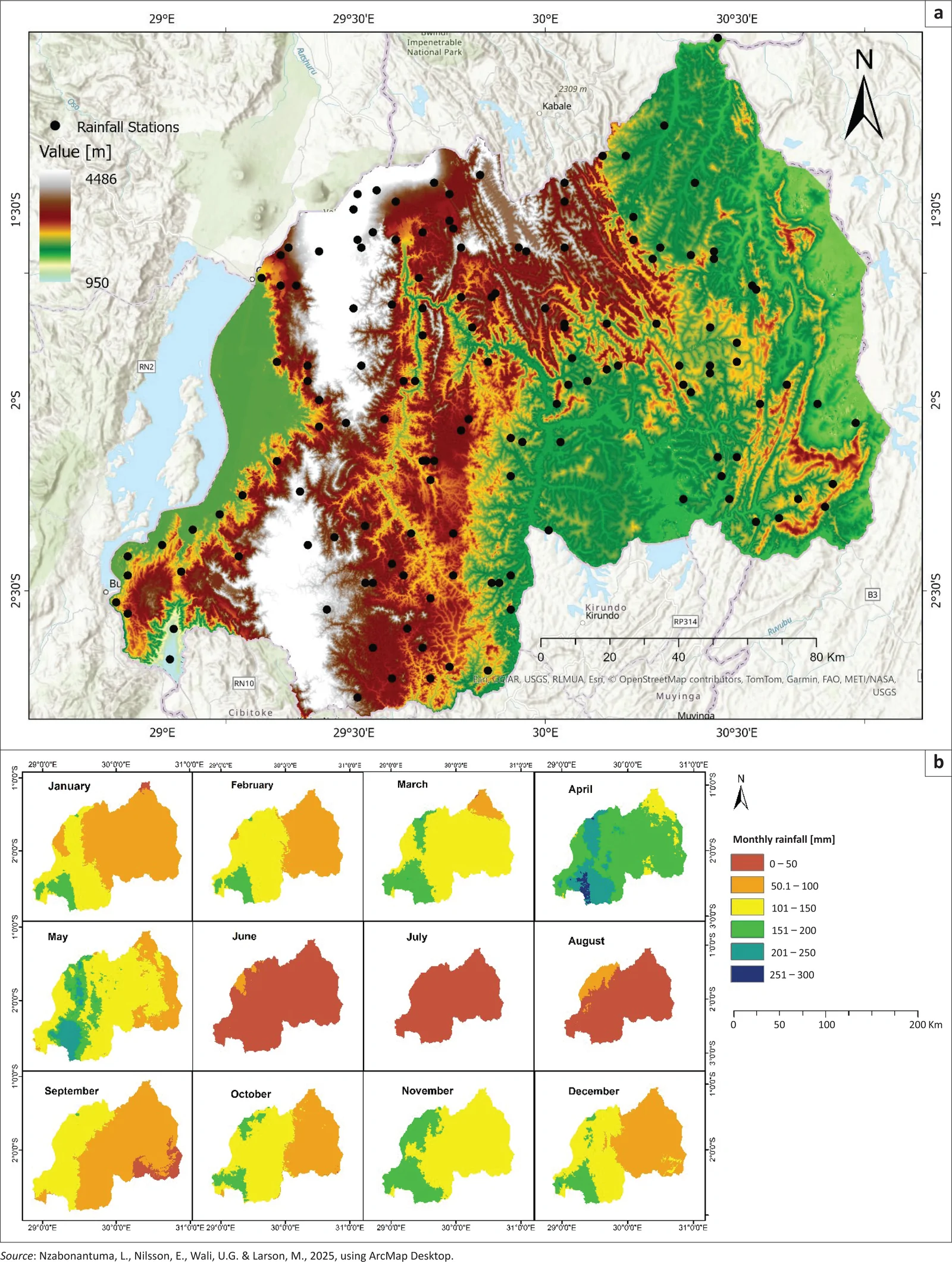
(a) Elevation map of Rwanda including the distribution of rain gauge stations up to 2021, and (b) monthly rainfall distribution across Rwanda in 2021.

The literature reveals a complex relationship between slope, stream density, elevation and flood risk (Dung et al. 2020). While steeper slopes can lead to the formation of more streams, potentially increasing stream density within a watershed, this does not necessarily equate to a higher flood risk. In fact, steeper slopes typically facilitate faster runoff and reduce water accumulation, often mitigating local flood risks in high-elevation areas. However, in lower-elevation regions, higher stream densities are generally associated with increased flood risk, as these areas can rapidly accumulate and channel water during heavy rainfall. This interplay of factors contributes to frequent flood events in Rwanda’s valleys and plains. Notable examples include recurrent flooding in areas such as the Sebeya catchment, Nyamutera along the Mukungwa River and the mouth of the Giciye River before it joins the Mukungwa River. Understanding these interactions is crucial for developing effective flood risk assessment, mitigation and management strategies (Schumann 2011).

#### Anthropogenic factors

Over the past nine decades, Rwanda’s population has surged with a nearly eightfold increase, from 1 595 400 people in 1934 to 15 200 000 in 2022 (NISR 2023). This rapid growth has exerted immense pressure on land, forests and associated ecosystems (Idukunda et al. 2025b). The expansion of agricultural and residential areas has led to extensive deforestation, reducing the ability of trees and soil to absorb and store water (Kayitesi et al. 2022). Forests in Rwanda are vital for regulating water flow, as they absorb excess rainfall and release it gradually, mitigating flash floods. Due to a landscape of steep slopes in Rwanda, tree roots are especially important to stabilise soil on steep slopes, preventing erosion and landslides that exacerbate flooding. Furthermore, forest cover reduces surface runoff, lowering flood risks downstream. Between 1960 and 2000, Rwanda lost approximately 65% of its natural forest area, shrinking from 634 000 to 221 000 hectares. This deforestation is largely driven by the country’s reliance on subsistence agriculture, which employs about 90% of the population (Ntabakirabose et al. 2024). Agricultural practices such as overgrazing, monoculture farming, poor land planning and insufficient soil conservation measures like contour ploughing and agroforestry have contributed to soil compaction and reduced vegetation cover (Karamage et al. 2016). These factors increase the surface runoff and accelerate erosion, and the resulting sediment deposition in streams heightens flood susceptibility (Kulimushi et al. 2021).

Illegal mining activities further aggravate flooding risks by exposing bare soil during land clearing, discharging contaminated wastewater into waterways and abandoning sites without proper rehabilitation. These practices lead to severe sedimentation in river systems and environmental degradation (Akayezu et al. 2020; REMA 2019).

Urbanisation in Rwanda has accelerated rapidly in recent decades, driving both opportunities and challenges. Between 2002 and 2015, Rwanda’s built-up area doubled from 83 km^2^ to 167 km^2^, while the urban share of the population grew from 15.8% to 26.5%, an increase of 132%, or almost 2 million people (The World Bank 2017). Moreover, projections indicate that 50% of Rwanda’s population will reside in urban areas by 2035 and increase to 70% by 2050 (Ministry of Environment (MoE 2022a). Kigali exemplifies this growth: its population surged from 358 200 in 1996 to 1 630 657 in 2017 and reached 1 745 555 by 2022 (NISR 2023). This trend mirrors broader urbanisation across the country, with other cities experiencing similar dynamics (Nduwayezu et al. 2021).

Rapid urbanisation and high population growth, particularly in Kigali City, have driven the expansion of informal settlements on steep and environmentally sensitive slopes, often lacking adequate stormwater management systems. This unplanned urban growth has also intensified surface runoff and heightened flood risk, as permeable natural surfaces are increasingly replaced by impervious structures such as buildings, roads and other grey infrastructure (Icyimpaye, Abdelbaki & Mourad 2022; Izere et al. 2024).

Severe flooding events in urban areas have clearly illustrated these factors and challenges. In December 2022, intense rainfall triggered flash floods that inflicted significant damage on property, infrastructure and livelihoods, particularly in Nyabugogo a densely populated market in Kigali City that remains highly susceptible to future flooding (Niyomugabo et al. 2022).

Despite these challenges, urbanisation in Rwanda has also contributed to economic improvements and poverty reduction. For example, a 10% increase in population density at the sector level has been associated with a 1.2% decrease in multi-dimensional poverty. Urban areas have further driven 48% of the country’s labour productivity growth over a 15-year period, boosting the average GDP per worker, and there are strong links between urban density and non-farm job creation, especially in Greater Kigali and other major cities (The World Bank 2017).

### Institutional framework

Since the early 2000s, Rwanda has undergone significant legal and institutional transformations to manage its water resources, environment and climate challenges. The institutional framework is designed to shift from fragmented management to a coordinated, sustainable approach integrated with national development goals like Vision 2050 and the National Strategy for Transformation Two (NST 2) (National Council for Research in Science and Technology [NCRST] 2023). The modern framework began with the 2004 water and sanitation policy, which initiated legal and political reforms in the sector. Initially, the MINIRENA was tasked with formulating water resources management policies, strategic planning and coordination. To provide a legal basis for these efforts, the government enacted Law N°62/2008, which regulated the use, conservation and protection of water resources. Despite these early steps, the framework initially lacked a central coordinating mechanism. This was addressed in 2010 with the establishment of the Rwanda Natural Resources Authority (RNRA). Furthermore, the Ministry of Infrastructure became a key stakeholder in policy development, particularly regarding water supply and sanitation services.

A cornerstone of Rwanda’s environmental framework is REMA, originally established in 2006. Its role was strengthened by Law no. 63/2013, which granted the agency enhanced autonomy and defined its mission to implement national environmental policies. Rwanda Environment Management Authority is responsible for advising the government on environmental strategies, ensuring compliance with laws through inspections and monitoring and providing technical support for both national and international collaborations (REMA 2019).

In 2020, the government established the Rwanda Water Resources Board (RWB) through Law N° 71/2019 to provide specialised focus on water sustainability. The RWB’s mandate includes: (1) advising the government on all water resource matters; (2) implementing strategies for flood and erosion control; (3) managing catchment protection and infrastructure development; (4) enforcing efficient water use and managing water-related data. The RWB operates collaboratively with REMA and the Water and Sanitation Corporation (WASAC) (Global Water Leadership Rwanda Programme 2024). Rwanda has moved towards a catchment-based management model to decentralise and improve water governance. Legal mandates followed, including Law No. 49/2018, which required the establishment of catchment management committees. In 2022, Ministerial Order No. 002/MoE/22 further operationalised this by creating catchment-level water resources management committees and a technical committee on dams (MoE 2022b). These catchment committees are chaired by district vice mayors in charge of economic affairs and operate under the RWB. However, these committees face practical hurdles, such as overlapping mandates, limited technical expertise and a continued reliance on external consultants for management plans (MoE 2022b).

Disaster and climate management are integrated through MINEMA and Meteo-Rwanda. Meteo-Rwanda provides critical weather and climate data to support socio-economic development and operates the Rwanda Climate Change Observatory (Global Water Leadership Rwanda Programme 2024). Disaster and climate management are integrated through MINEMA and Meteo-Rwanda. Meteo-Rwanda provides critical weather and climate data to support socio-economic development and operates the Rwanda Climate Change Observatory. The disaster management framework has evolved from the 2012 National Disaster Management Policy to the revised 2023 policy (MINEMA 2023b). The revision was necessary because the original framework had vague stakeholder roles, leading to the duplication of efforts and the exclusion of key research and water management institutions. The current disaster management structure is illustrated in Table 2 and Figure 5.

**FIGURE 5:**
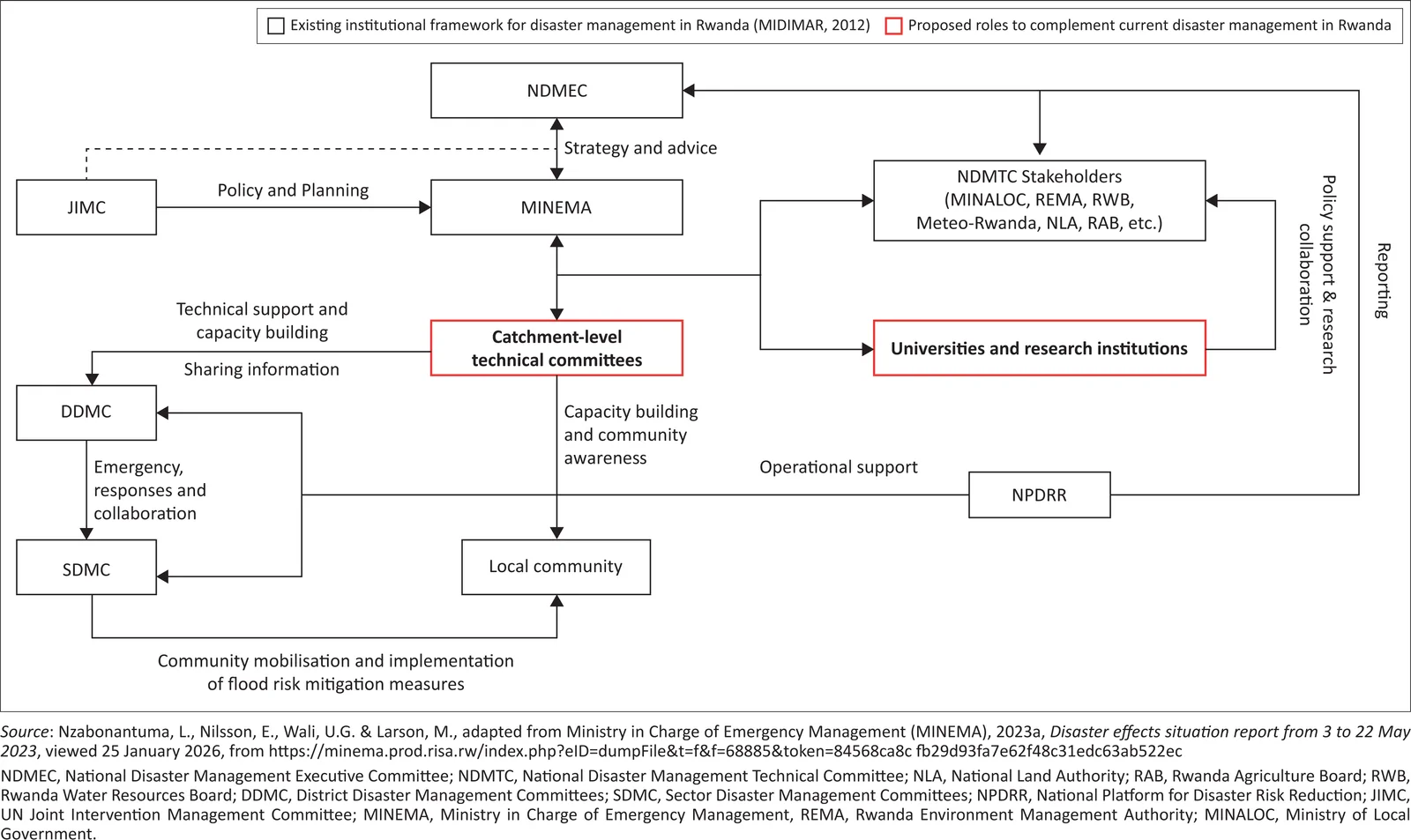
Proposed framework for flood risk management in Rwanda.

To support evidence-based decision-making, the government has integrated research institutions and universities into its policy frameworks (MINEMA 2023). The National Council of Science and Technology (NCST) promotes research through programmes like ‘Grand Challenges Rwanda’, which provides grants for innovation in national development (NCRST 2023).

Despite these institutional strengths, significant challenges remain at the community level. Many citizens perceive flood mitigation as a sole government responsibility, leading to a lack of shared accountability. There is also a lack of public knowledge regarding early warning systems and emergency preparedness. Furthermore, some disaster management agencies remain inactive or ineffective due to a lack of functional capacity, and financial constraints continue to leave low-income groups vulnerable to hazards (Mind’je et al. 2019; MINEMA 2023b). The entire institutional framework is designed to align with Vision 2050 and NST 2. These overarching strategies prioritise a green economy, infrastructure investment and the mainstreaming of Disaster Risk Reduction (DRR) across all sectors. The mandate of the RWB reflects Rwanda’s commitment to achieving high-income status through sustainable environmental protection and climate resilience (Government of Rwanda [GoR] 2024; MINEMA 2023b; Perez-Guzman et al. 2023).

### Implemented measures

While the review of the institutional framework shows that there is a lack of specific FRM legislation, Rwanda has proactively implemented a diverse range of FRM measures. These efforts are largely driven by REMA and RWB, focusing on a combination of nature-based solutions (NbS), structural engineering and strategic planning. Rwanda has increasingly positioned itself as a regional leader in the adoption and implementation of NbS, with a strong emphasis on integrating both urban and rural resilience strategies (REMA 2019). In this regard, comprehensive catchment management plans have been developed and implemented for the Sebeya, Upper Nyabarongo, Nyabugogo and Muvumba catchments (REMA 2020). These initiatives include, among other interventions, large-scale reforestation on steep and erosion-prone hillsides, as well as the ‘Scaling Urban Nature-based Solutions’ project, which aims to plant approximately 2 million trees between 2024 and 2027, including about 125 000 fruit trees to enhance both ecological and livelihood benefits (Uwacu et al. 2021). In addition, projects funded by the Least Developed Countries Fund II (2017–2022) have focused on restoring degraded wetlands and forest ecosystems, while simultaneously supporting local communities through the provision of livestock as a means of strengthening adaptive capacity and livelihood resilience.

Terracing has been widely adopted as a primary land management strategy for controlling surface runoff and reducing soil erosion in Rwanda’s hilly landscapes. By 2018, approximately 26% of erosion-prone land had been protected through the construction of terraces (RWB & International Union for Conservation of Nature [IUCN] 2022). Empirical research further demonstrates that bench terraces can reduce soil erosion by up to 100% in specific agro-ecological zones, while radical terraces can reduce erosion by about 90% when adequately constructed and properly maintained (Rutebuka et al. 2021). Collectively, these measures play a critical role in stabilising slopes, limiting sediment transport and reducing siltation processes that exacerbate downstream flooding.

Complementing these nature-based interventions, the GoR has also invested in grey and hybrid infrastructure to improve stormwater management and regulate river levels. For instance, in Musanze District, drainage systems have been widened and detention basins constructed to enhance runoff retention, while along the Sebeya River, flood risk is mitigated through the construction of dykes and additional detention basins designed to manage stormwater more effectively (Hahirwabasenga et al. 2024).

The RWB’s 2021–2030 strategic plan focuses on enhancing resilience through preparedness, prevention and the identification of high-risk zones. To ensure long-term sustainability, Rwanda has initiated an environmental education strategy that includes ‘greening’ schools and capacity building. However, a current limitation is the strategy’s focus on secondary schools, which may miss the larger population of students in primary education. Collectively, these measures emphasise a shift towards proactive mitigation and climate adaptation (Nahayo et al. 2018; Priest et al. 2016).

### Availability of data

Flood risk management in Rwanda is fundamentally dependent on the availability, quality and integration of diverse datasets that support evidence-based decision-making. These datasets are primarily managed by two key institutions, RWB and Meteo-Rwanda, which play crucial roles in hydrological monitoring and climate information dissemination. Although the country has made notable progress in developing early warning systems and adopting remote sensing technologies, substantial challenges remain, particularly regarding persistent data gaps and limited monitoring infrastructure.

From a historical perspective, Rwanda’s data collection capacity has undergone significant fluctuations. During the 1960s, the country operated a relatively dense network of 147 rain gauges. However, this infrastructure declined sharply in the aftermath of the 1994 genocide against the Tutsi, leaving only 11 operational rain gauges by 2006 (Ndekezi 2012). In recent years, the national observation network has gradually expanded to include both manual and automatic weather stations, as well as radar-based information that is now accessible through an online data portal.

Nevertheless, the spatial density of these stations remains far too low to enable comprehensive flood modelling or provide reliable real-time monitoring in remote and flood-prone catchments.

Current research on FRM in Rwanda tends to focus heavily on the physical drivers of flooding. A review of published studies shows that hydro-meteorological data are used most frequently (45%), followed by topographic data (38%) and Land-use and land cover information (36%) (Table 1). Due to limitations in ground-based monitoring, many researchers rely extensively on remote sensing and model-based approaches to supplement climate and precipitation datasets. In contrast, hydraulic data, vital for capturing river channel geometry and conveying flow characteristics, are employed in only about 14% of studies, which restricts the development and validation of robust hydraulic models.

**TABLE 1:** Data used in reviewed research publications.

Data category	Data types	Typical data sources	Number of publications	
			*n*	%
Hydro-meteorological	Rainfall (daily and/or seasonal), rainfall trends, streamflow, peak flows	Rain gauges, hydrometric stations, CHIRPS, TRMM, reanalysis	19	45
Climate	ENSO, SSTs, IOD, atmospheric circulation variables	Global climate datasets	8	19
Topography & terrain	DEM, elevation, slope, flow accumulation, watershed boundaries	SRTM, ASTER, GIS-derived layers	16	38
Hydraulic	River channel geometry (cross-sections, width, depth), conveyance characteristics, roughness proxies	Field surveys, DEM-derived channel metrics	6	14
Surface water	Flood inundation extent, river and wetland water masks	Sentinel-1 SAR, NDWI/MNDWI, GIS mapping	8	19
Land use/land cover	LULC classes, urban expansion, imperviousness proxies	Landsat, Sentinel-2, national LULC, RF/U-Net, GEE	15	36
Vegetation cover	NDVI, vegetation cover indicators	Landsat, Sentinel-2	7	17
Soil properties	Soil texture, depth, erodibility, infiltration proxies	FAO soils, national surveys, field sampling	11	26
Erosion & sediment	Sediment yield, soil loss, erosion hotspots, sediment fingerprints	Field/lab data, USLE/RUSLE, remote sensing-derived proxies	10	24
Infrastructure & settlements	Buildings, roads, drainage, informal settlements	GIS layers, local authority data	9	21
Demography	Household data, population, livelihoods	Surveys, census, administrative data	13	31
Risk perception	Awareness, preparedness, coping strategies	Interviews, questionnaires	8	19
Flood impact	Flood damages, losses, fatalities	Disaster impact records, surveys, secondary reports	8	19
Policy & institutional	Laws, strategies, governance arrangements	Government documents, policy reports	9	21

Note: Please see full reference list of Nzabonantuma, L., Nilsson, E., Wali, U.G. & Larson, M., 2026, ‘Flood risk management in Rwanda: A review of basic conditions and recommendations for an enhanced institutional framework’, *Jàmbá: Journal of Disaster Risk Studies* 18(1), a2102. https://doi.org/10.4102/jamba.v18i1.2102

ENSO, El-Nino-Southern Oscillation; IOD, Indian Ocean Dipole; DEM, digital elevation model; SST, sea surface temperature; CHIRPS, Climate Hazards Group InfraRed Precipitation with Station data; TRMN, Tropical Rainfall Measuring Mission; SRTM, Shuttle Radar Topography Mission; ASTER, Advanced Spaceborne Thermal Emission and Reflection Radiometer; GIS, Geographic Information System; SAR, Synthetic Aperture Radar; NDWI/MNDWI, Normalized Difference Water Index and Modified Normalized Difference Water Index; LULC, Land-use and Land Cover; RF/U-Net, Random Forest and U-shaped convolutional neural network architecture; Google Earth Engine; Normalized Difference Vegetation Index; USLE/RUSLE, Universal Soil Loss Equation and Revised Universal Soil Loss Equation.

Beyond hydrological parameters, significant data gaps remain in flood impact information, while evidence on community flood risk perception is still limited, indicating the need for more comprehensive data collection and monitoring (Hahirwabasenga et al. 2024). Although MINEMA publishes reports documenting the frequency and distribution of flood events, these reports often combine multiple hazards such as landslides and intense rainstorms, making it difficult to disentangle the impacts of floods specifically (MINEMA 2024a). Moreover, socio-economic data are frequently derived from localised surveys, which often differ in methodology and lack standardisation across administrative regions (Habumugisha & Mukashyaka 2023; Hahirwabasenga et al. 2024).

In recent years, however, Rwanda has made commendable operational improvements. Notably, the development of a flood early warning system in the Sebeya River Basin and the publication of the *Compendium of Environment Statistics* by REMA have strengthened national capacity for environmental monitoring (Icyimpaye et al. 2022). Despite these advances, persistent challenges related to data quality such as recording errors, missing values and temporal discontinuities, continue to affect reliability. Furthermore, data management responsibilities remain fragmented across multiple agencies, including METEO-Rwanda, MINEMA, RWB and REMA. This fragmentation, coupled with the absence of a unified data-sharing platform, means that researchers and practitioners must often submit direct requests to access relevant datasets. Consequently, the limited openness and irregular availability of data continue to hinder the efficiency and reproducibility of hydrological and hydraulic modelling efforts (Mind et al. 2020).

## Discussion

### Key issues and challenges

Rwanda faces escalating flood risks characterised by increased frequency, rising casualties and substantial economic losses that hinder national development and GDP growth. These challenges are driven by a combination of environmental, human and institutional factors.

The primary environmental triggers include steep terrain and high rainfall in mountainous regions, which are increasingly worsened by climate change. These are compounded by human activities such as deforestation, unregulated mining, rapid, unplanned urbanisation and intensive agriculture with poor or unsustainable practices, all of which degrade catchments and increase runoff and soil erosion (REMA 2021). While measures like reforestation and terracing have been introduced, they currently lack the scale and continuity required for long-term impact.

A critical issue is the absence of a dedicated FRM policy; instead, flood risk is managed under broader disaster management frameworks, which may lack specificity (Habumugisha & Mukashyaka 2023; Idukunda et al. 2025b; MINEMA 2023b). Furthermore, management is frequently organised by administrative boundaries rather than hydrological catchments, leading to a coordination disconnect. The catchment committees often lack the necessary technical expertise and clear responsibilities, frequently relying on short-term external contracts rather than stable, community-led expert committees. This fragmentation is worsened by limited institutional capacity within agencies like MINEMA and a lack of stakeholder coordination.

Technical weaknesses include data scarcity and deficiencies in early warning systems, which weaken disaster preparedness. As noted in previous discussions, the historical decline of monitoring infrastructure and the lack of integrated data platforms remain significant hurdles.

Additionally, financial constraints and a high socio-economic dependence on agriculture increase vulnerability (Mwenje & Kumar 2024). Finally, educational outreach is currently too narrow, failing to provide early engagement at the primary school level, which limits public awareness and community engagement in mitigation efforts (MINEMA 2023b).

### Proposal for improved flood risk management

#### Institutional framework

To enhance FRM in Rwanda, the review proposes a shift towards adaptive governance principles, drawing from international models such as the European Union Floods Directive (Djalante, Holley & Thomalla 2011; Priest et al. 2016). Adaptive governance recognises that flood risk is characterised by uncertainty, complexity and changing climatic conditions, and therefore requires flexible, collaborative and learning-oriented institutional arrangements operating across multiple governance levels.

A central tenet of this proposal is the principle of subsidiarity, which asserts that governance responsibilities should be assigned to the lowest practical level of authority capable of addressing them, while higher levels provide support for cross-scale challenges (Priest et al. 2016).

Accordingly, community-level institutions remain the primary actors responsible for local flood preparedness, early warning dissemination, emergency response, implementation of locally appropriate risk reduction measures and communication of community priorities and indigenous knowledge. Higher-level institutions should complement, not replace, these local arrangements by addressing functions requiring broader technical expertise, inter-jurisdictional coordination and strategic planning.

This principle is complemented by a ‘designing for failure’ philosophy, which advocates for a multi-layered strategy rather than relying on a single intervention (Schumann 2011). Such an approach is vital for managing residual risk, the risk that remains when existing protections, such as the Sebeya River dykes, fail due to extreme events or climate variability (Idukunda et al. 2025a; Priest et al. 2016; RWB 2023). Effective FRM therefore requires coordinated investments in structural measures, land-use planning, ecosystem-based solutions, preparedness, early warning systems and community resilience.

Within this governance framework, the review proposes establishing Catchment-Level Technical Committees (CLTCs) as coordination and technical advisory bodies operating at the river catchment scale (Figure 5; Table 2). Importantly, these committees are not intended to replace existing community disaster risk management (DRM) committees, village structures or district disaster management arrangements, but rather to complement them by providing scientific and technical support for issues that extend beyond administrative boundaries. Whereas existing district disaster management committees, often chaired by vice-mayors, are primarily administrative and emergency-response oriented, the proposed CLTCs would strengthen the technical dimension of flood risk governance through multidisciplinary expertise in hydrology, hydraulic engineering, environmental management, spatial planning, socioeconomics and climate adaptation.

**TABLE 2:** Description of organisations and responsibilities in proposed framework to enhance flood risk management in Rwanda.

Acronym	Name	Members	Description of responsibilities
NDMEC	*National Disaster Management Committee*	Minister of Ministry of Emergency (MINEMA), Minister of Defence, Minister of Internal Security, Minister of Local Government, Minister of Health, Minister of Infrastructure, Minister of Finance and Economic Planning, Minister of Agriculture and Animal Resources, Minister of Natural Resources, Chief of Defence Staff, and Inspector General of Police.	(1) Policy guidance, strategic advice, and coordination (2) Sharing information and technical expertise nationally, regionally and internationally; (3) reviewing policies and relevant plans; (4) ensuring a multi-stakeholder participation in the development, updating, and sharing of Disaster Risk Reduction and Management Information System; (5) developing and implementing the national strategy for disaster risk management; (6) monitoring and analysis of hazard risk trends in the; (7) developing common preparedness, contingency and response plans with relevant public institutions in Rwanda.
MINEMA	Ministry of Emergency		(1) Coordinating disaster risk management activities across government agencies, NGOs, civil society, and humanitarian actors; (2) conducting policy development, planning and coordination.
NDMTC	National Disaster Management Technical Committee	All Ministries are part of the National Disaster Management Executive Committee appoints a permanent focal point in the National Disaster Management Technical Committee. Universities and research institutions will be part of NDMTC	(1) Planning, developing, and implementing policies and programmes related to disaster management; (2) ensuring technical advice; (3) overseeing with public institutions for decision implementation, and (4) supporting decision-making through research activities.
JIMC	Joint Intervention Management Committee	The Joint Intervention Management Committee consists of representatives at the decision-making level of MINEMA and relevant agencies of the United Nations. The private sector will be represented.	Facilitating the coordination between MINEMA and the United Nations in Rwanda and the private sector.
CLTCs	Catchment-Level Technical Committees (CLTCs)	A multidisciplinary technical team appointed by MINEMA in collaboration with RWB and relevant sector institutions, comprising experts in hydrology, hydraulic engineering, environmental management, spatial planning, climate adaptation, disaster risk management and socio-economic analysis for each Level 1 river catchment.	(1) Collect, integrate, analyse and interpret near real-time hydrological, meteorological and environmental data to support flood forecasting and early warning; (2) conduct flood hazard, exposure, vulnerability and risk assessments at the catchment scale; (3) prepare, coordinate, periodically review and update catchment-level Flood Risk Management (FRM) plans; (4) provide scientific and technical guidance to District Disaster Management Committees (DDMCs), Sector Disaster Management Committees (SDMCs), and community-level DRM structures; (5) coordinate upstream-downstream flood mitigation, watershed restoration and integrated catchment management interventions across multiple districts; (6) monitor, evaluate and provide technical oversight of catchment-wide structural, non-structural and Nature-based Solution (NbS) interventions; (7) translate scientific information into practical guidance for local authorities and communities through capacity building, awareness programmes and technical support; (8) facilitate collaboration among government institutions, universities, research organisations, civil society and the private sector to strengthen evidence-based flood risk management; (9) promote integrated data sharing, hydrological modelling, and adaptive flood risk governance across institutions; and (10) report technical findings, recommendations and catchment performance to MINEMA, RWB and the National Disaster Management Executive Committee (NDMEC), while supporting continuous policy improvement and adaptive management.
DDMC	District Disaster Management Committees	(1) District Mayor, (2) District Army Commander, (3) District Police Commander, (4) Professional in Charge of Disaster Management in the District, (5) District Social protection officer, (6) Director of the district hospital, (7) Rwanda Red Cross District Representative.	
SDMC	Sector Disaster Management Committees	(1) Sector Executive Secretary, (2) Sector Social Affairs Officer, (3) Reserve force commander, (4) Sector army commander, (5) Sector Police Commander, (6) Local Defence Force Commander in the Sector, (7) Head of Community Health Workers, (8) Sector Rwandan Red Cross Representative	(1) Collecting early warning information and transmitting it to the CMCC and district level; (2) collecting and disseminating disaster information; (3) planning and mobilising committees in decision-making; (4) strengthening local authorities in disaster management; (5) implementing interventions; (6) conducting community mobilisation and awareness campaigns.

*Source*: Nzabonantuma, L., Nilsson, E., Wali, U.G. & Larson, M., adapted from Ministry in Charge of Emergency Management (MINEMA), 2023b, National disaster risk reduction and management policy, Rwanda, viewed 25 January 2026, from https://www.minema.gov.rw/index.php?eID=dumpFile&t=f&f=70104&token=1e6bdd5b22ad6a5455e3c313753ea76b327568a3

Note: Please see full reference list of Nzabonantuma, L., Nilsson, E., Wali, U.G. & Larson, M., 2026, ‘Flood risk management in Rwanda: A review of basic conditions and recommendations for an enhanced institutional framework’, *Jàmbá: Journal of Disaster Risk Studies* 18(1), a2102. https://doi.org/10.4102/jamba.v18i1.2102

NGO, non-governmental organisation; RWB, Rwanda Water Resources Board; DRM, disaster risk management.

Operating at the hydrological catchment scale would enable these committees to address one of Rwanda’s major institutional challenges: the fragmentation of flood management across district administrative boundaries despite floods occurring within interconnected river systems. Their primary responsibilities would include: (1) collecting, integrating and analysing near real-time hydrological and meteorological data; (2) conducting flood hazard, vulnerability and risk assessments; (3) preparing, coordinating and periodically updating catchment-level FRM plans; (4) providing technical guidance to districts and community-level DRM structures; (5) coordinating upstream–downstream interventions across multiple districts; (6) monitoring implementation of catchment-wide mitigation measures; and (7) reporting technical findings and recommendations to RWB, MINEMA and the National Disaster Management Executive Committee.

Consistent with the principle of subsidiarity, the interface between CLTCs and community-level institutions is collaborative rather than hierarchical. Community DRM committees and other local institutional arrangements would continue leading community mobilisation, awareness campaigns, evacuation planning, local monitoring of flood-prone areas, maintenance of community-based mitigation measures and dissemination of early warning information. These local institutions would also communicate local knowledge, observed flood impacts and community priorities to district authorities and the CLTCs through established reporting mechanisms and regular stakeholder forums. In turn, the CLTCs would translate scientific information into locally relevant technical guidance, support community risk assessments, strengthen local capacity through training and ensure that community concerns inform catchment-scale planning. This two-way exchange creates a continuous feedback mechanism, whereby scientific evidence and local knowledge jointly inform adaptive FRM.

Implementation of the proposed framework should follow a phased approach, initially prioritising high-risk Level 1 catchments to build institutional experience, refine operational procedures and demonstrate effectiveness before wider national implementation. Throughout this process, MINEMA would retain overall responsibility for national policy, strategic coordination, standard setting, resource mobilisation and monitoring, while the CLTCs would coordinate technical planning at the catchment level and district, and community institutions would remain responsible for implementation of locally appropriate risk reduction and disaster preparedness activities.

The proposed framework also recognises that technical expertise alone is insufficient for effective FRM. Sustainable FRM requires an integrated governance system combining multidisciplinary scientific expertise with institutional coordination, stakeholder participation, spatial planning, ecosystem management, effective policy instruments and continuous learning. In line with the revised National Disaster Management Policy (MINEMA 2023b), MINEMA would therefore play a central coordinating role, ensuring coherence among sectoral institutions while promoting collaboration across national, catchment, district and community levels.

Building on this adaptive governance framework, institutional reforms should also strengthen upstream–downstream coordination within catchments. In particular, upstream landowners and local authorities should assume explicit responsibility for implementing runoff control measures, watershed conservation and sustainable land management practices that reduce downstream flood risk (Sujono 2010). To make this transition effective, continuous community sensitisation and capacity-building efforts are needed, along with targeted incentives that support local authorities in demonstrating best practices and success stories.

Finally, universities and research institutions should become integral partners within the proposed governance framework. Through collaboration with government agencies, local authorities, civil society and the private sector, these institutions can generate evidence-based knowledge, improve hydrological modelling and flood forecasting, evaluate policy effectiveness and support innovation in FRM (MINEMA 2023b). Their contributions would strengthen Rwanda’s technical capacity while supporting the development of a proactive, adaptive and catchment-based FRM policy. Collectively, these institutional reforms would help transform Rwanda’s flood management system from a predominantly reactive disaster response model towards a proactive, coordinated and scientifically informed flood risk governance framework (MINEMA 2023b; RWB 2023).

#### Mitigation measures

To strengthen FRM in Rwanda, recent sources advocate for a broad and integrated suite of mitigation measures that encompass NbS, urban infrastructure adaptations and socio-economic strategies. Collectively, these interventions aim to address persistent challenges, including the fact that only about 26% of erosion-prone land in the country is currently protected through terracing (MINEMA 2024b; RWB & IUCN 2022; Uwacu et al. 2021). This gap highlights an urgent need for more coordinated and scientifically informed land and water management approaches.

Expanding the implementation of terracing stands out as a central recommendation within the rural context. Terraces not only enhance soil and water retention, but also promote groundwater recharge while simultaneously improving agricultural productivity (Rutebuka et al. 2021). To further optimise their effectiveness, several studies recommend combining terracing with the construction of trenches or diversion ditches along terrace contours, which can intercept surface runoff and trap sediments before they reach and silt downstream channels (Lafevor & Ramos-Scharrón 2021).

For river and hillside restoration, three key interventions are prioritised.(1) The installation of check dams across small stream channels, coupled with the strategic planting of vetiver grasses such as *Brachiaria* and *Pennisetum purpureum* along stream banks, helps slow water velocity, increase infiltration and enhance channel stability (Zhang et al. 2022). These interventions provide triple benefits to local communities: they reduce soil erosion, improve water availability and supply fodder for livestock, thereby motivating local farmers to maintain and sustain these measure; (2) Promoting more sustainable agricultural methods, particularly contour ploughing, no-till planting and maintaining crop residue cover, can substantially reduce surface runoff and minimise topsoil erosion on cultivated hillsides; and (3) Establishing vegetated buffers between farmlands and waterways is identified as a particularly cost-effective measure for sediment control (Kagabo et al. 2013; Neachell 2014). In addition, scaling up agroforestry systems, with clear technical guidance on species selection, spacing and row orientation, is viewed as a vital step towards strengthening environmental resilience and enhancing long-term soil fertility.

In urban settings, the focus shifts towards integrated stormwater management and infrastructure adaptation to minimise flood risks at the source (MoE 2022a). The promotion of green roofs offers multiple benefits, including reducing stormwater runoff volumes and mitigating the urban heat island effect (Ferreira et al. 2022). When coupled with rainwater harvesting systems, these measures not only alleviate pressure on drainage networks, but also contribute to local water storage and reuse. The reviewed sources further emphasise the need for expanded use of retention and detention basins, particularly in rapidly growing urban centres such as Kigali, Musanze, Huye and Mahoko. Complementary measures, including the development of rain gardens, constructed wetlands and rice paddies, can act as effective natural storage systems that delay and attenuate peak runoff (Freni & Liuzzo 2019).

Finally, the introduction of flood insurance mechanisms is recommended to safeguard vulnerable households and manage the economic consequences of residual flood events. When effectively combined, these technical, institutional and financial measures, anchored under the guidance of expert-led CLTCs, could enable Rwanda to shift from a predominantly reactive approach to flood response towards a proactive, integrated and multi-layered management framework capable of anticipating and mitigating flood risks more sustainably.

#### Data and monitoring

Literature on FRM in Rwanda underscores a progressive transition towards establishing a more robust and high-resolution data monitoring framework. Such a transformation is essential not only to address the long-standing decline in hydrometric infrastructure, but also to strengthen the country’s capacity for accurate forecasting and timely flood response. In this context, the creation of a resilient and integrated data ecosystem emerges as a central pillar for sustainable FRM (Abimbola et al. 2017; Hahirwabasenga et al. 2024; RWB 2021).

A key priority identified by multiple sources is the need to substantially increase the spatial density of rainfall and stream gauging stations, particularly within flood-prone and rapidly urbanising catchments such as Sebeya and Nyabarongo. To enable more precise event-based modelling, there is a growing call to move beyond traditional daily observations and adopt higher-frequency (hourly) data collection facilitated by automated, telemetry-enabled sensors. These enhanced ground-based observations should be systematically integrated with remote sensing products and high-resolution Digital Elevation Model (DEM) to improve the accuracy of hydrological assessments in data-scarce and topographically complex regions. In addition, emerging innovations such as motion-activated cameras installed at strategic sites, including bridge crossings, offer promising opportunities for real-time and dynamic impact assessments (Macharia et al. 2023).

However, technological improvements alone are insufficient without corresponding institutional and human capacity development. Therefore, targeted capacity-building initiatives within key national agencies, particularly Meteo-Rwanda and RWB, are crucial for enhancing database management, data quality assurance and advanced hydraulic modelling capabilities (Macharia et al. 2023). Equally important is the establishment of centralised and user-friendly data-sharing platforms designed to facilitate transparency, interoperability and broader knowledge exchange across government departments, researchers and development partners. In alignment with the subsidiarity principle highlighted in earlier discussions, the literature also emphasises the integration of local communities into data collection and disaster education initiatives. Actively involving local stakeholders ensure that community-based knowledge and experiential insights inform national decision-making processes. At the same time, institutional policies must continue to evolve to promote open access, collaborative data management and adaptive learning across administrative levels. Ultimately, the implementation of such comprehensive and inclusive monitoring measures is indispensable for strengthening Rwanda’s flood early warning systems and for designing effective, evidence-based mitigation strategies that protect both people and infrastructure.

#### Research

Improved monitoring and data availability will enable the research sector to take deliberate and proactive steps to advance FRM in Rwanda. Drawing on insights from recent studies and ongoing projects, the following research priorities have emerged as particularly critical for moving FRM efforts forward: (1) investigating the drivers and impacts of floods in key catchments; (2) enhancing the accuracy and reliability of hydrological and flood hazard modelling; (3) assessing the socio-economic and institutional dimensions that influence vulnerability and resilience; (4) optimising early warning systems to ensure timely and effective responses; (5) exploring the potential synergies between grey and green infrastructure solutions; and (6) facilitating the exchange of best practices with neighbouring countries, while also expanding technical assistance from global partners such as the World Bank and the European Union, particularly in piloting innovative flood monitoring tools. To support and sustain these efforts, the GoR should promote FRM research by formalising research collaborations within the proposed institutional framework, increasing funding and supporting the establishment of dedicated research institutions.

## Conclusion

Rwanda faces escalating flood risks driven by heavy rainfall, steep topography and rapid, unplanned urbanisation. While the country has established robust institutions like REMA and RWB and implemented significant measures such as terracing and reforestation, current efforts are hampered by fragmented data, limited technical capacity at local levels and the lack of a dedicated FRM policy. To safeguard national development and meet Vision 2050 goals, Rwanda must transition from a reactive disaster response to a proactive, catchment-based and technically grounded management framework. This study therefore recommends: (1) Replace administrative-led committees with expert bodies specialised in hydrology, hydraulic engineering, environmental management and socioeconomics to manage risks across administrative boundaries; (2) Increase the density of automated, telemetry-enabled gauging stations and adopt hourly data collection to support real-time early warning systems and accurate hydraulic modelling; (3) Prioritise NbS, such as combining terracing with diversion ditches in rural areas and implementing green urban infrastructure like retention basins and rain gardens in rapidly growing cities; (4) Create a unified data-sharing platform between agencies (Meteo-Rwanda, MINEMA, RWB, REMA) and formalise collaborations with universities to ensure evidence-based policy development; (5) Enhance subsidiarity by involving local communities in data collection and incentivising upstream landowners to take responsibility for sustainable runoff control; and (6) Move beyond general disaster frameworks to enact specific FRM legislation that clarifies stakeholder roles and mandates long-term catchment-level planning.

## APPENDIX 1

**TABLE 1-A1:** List of stakeholders.

No	Responsible institutions
1	Ministry of Disaster Management and Refugee Affairs (Lead)
2	Ministry of Natural Resources (co-lead)
3	Ministry of Local Government
4	Ministry of Agriculture and Animal Resources
5	Ministry of Defence
6	Ministry of Finance and Economic Planning
7	Ministry of Infrastructures
8	Ministry of Health
9	Rwanda Environment Management Authority
10	Rwanda Land Management and Use Authority
11	Rwanda Red Cross
12	Local Government
13	Rwanda Water Resources Board
14	METEO-Rwanda
15	National Institute of statistics
16	Rwanda Environment Management Agency (REMA)

*Source:* MINEMA (2023)

METEOx, METEO-Rwanda, Rwanda Meteorology Agency.

**TABLE 2-A1:** Summary of the publications included in the review.

Authors	Year	Title	Journal	Study area	Main findings
1. Kayitesi et al.	2022	Impact of land use land cover and climate change (LU/LCC) on river hydro-morphology – A review of research studies in tropical regions	*Journal of Hydrology*	Tropical areas	LU/LCC and hydrological variables including catchment heterogeneity control hydrology processes
2. Mind’je et al.	2019	Flood susceptibility modelling and hazard perception in Rwanda	*International Journal of Disaster Risk Reduction*	Rwanda	NDVI and rainfall are the most influencing variables for estimating flood risk, and lack of shared responsibilities between community members and government in FRM because of community disaster perception.
3. Jonah et al.	2021	Spatiotemporal variability of rainfall trends and influencing factors in Rwanda	*Journal of Atmospheric and Solar-Terrestrial Physics*	Rwanda	MAM (SOND) has a weak (strong) relationship with the Indian sea surface temperature (SST), while El-Nino Southern Oscillation (ENSO) influences strongly on September-December (SOND) rainy seasons than on March-May (MAM) rainy seasons.
4. Abimbola et al.	2017	The assessment of water resources in ungauged catchments in Rwanda	*Journal of Hydrology: Regional Studies*	Rwanda	Climate, physiography and land cover strongly influence the hydrology of the catchments in Rwanda. Using Leave-one out cross-validation, the log-transformed models were found to predict suitably the flow parameters and can be used to predict flow parameters in ungauged catchments in Rwanda.
5. De la paix et al.	2013	Soil degradation and altered flood risk because of deforestation	*Land Degradation and Development*	Rwanda	The use of fuelwood and competition for agriculture land are the main causes of deforestation, which leads to soil erosion and floods
6. Habiyaremye et al.	2011	Demographic pressure impacts on forests in Rwanda	*African Journal of Agricultural Research*	Rwanda	The effects of deforestation and its associated consequences are results of demographic pressure in Rwanda
7. Nones	2019	Dealing with sediment transport in FRM	*Acta Geophysica*	Italy	The consideration of sediment transport in drawing flood risk should be account for the dynamic behaviour of surface waters.
8. Kulimushi et al.	2021	Erosion risk assessment through prioritisation of sub-watersheds in Nyabarongo river catchment, Rwanda	*Environmental Challenges*	Rwanda	The effective soil conservation measures at a priority basis are needed for sustained productivity while minimising undesirable effects on the environment
9. Kagabo et al.	2013	Soil erosion, soil fertility and crop yield on slow-forming terraces in highlands of Buberuka, Rwanda	*Soil & Tillage Research*	Rwanda	Well established grass strips alone or combined with infiltration ditches are clearly effective at reducing erosion, and the gradually formed natural terraces between grass strips showed a marked spatial difference in both soil quality and crop yield from their upper parts downwards.
10. Ndekezi	2012	Hydrological modelling of Nyabugogo River basin in Rwanda using combinations of precipitation input from meteorological models, remote sensing and ground station measurements.	Book	Rwanda	Techniques to combine rainfall data sources and to produce rainfall datasets improve the quality of global rainfall data.
11. Icyimpaye et al.	2022	Hydrological and hydraulic model for flood forecasting in Rwanda	*Modeling Earth Systems and Environment*	Rwanda	In Nyabugogo river, flooding is caused by high rainfall intensity, topography, soil texture, urbanisation, informal settlement and inappropriate agriculture practices.
12. Mind et al.	2020	Landslide susceptibility and influencing factors	*Environment, Development and Sustainability*	Rwanda	This study used the Frequency Ratio model to map landslide susceptibility across Rwanda. Results identify the western, northern and southern provinces as the most high-risk areas. Primary drivers include slope, land use, rainfall and elevation. The model achieved an 84.6% prediction rate.
13. Akayezu et al.	2020	Using sediment finger printing to identify erosion hotspots in a sub-catchment of lake Kivu, Rwanda	*Environmental Monitoring and Assessment*	Rwanda	It was found that 70.9% of the Sebeya catchment area contributes at least 50% of sediment load in the river and currently experiences unsustainable land use and land cover
14. Nahayo et al.	2018	Extent of disaster courses delivery for the risk reduction in Rwanda	*International Journal of Disaster Risk Reduction*	Rwanda	There is a good level of disaster risk reduction awareness among respondents and related courses delivery rate is low in Rwanda
15. Priest et al.	2016	The European Union approach to FRM and improving societal resilience: lessons from the implementation of the Floods Directives in six countries	*Ecology and Society*	Europe	The research provides a legal and policy analysis of the implementation of the Floods Directive (2007/60/EC) in six countries: Belgium (Flemish region), England, France, the Netherlands, Poland, and Sweden, and evaluate criteria from existing legal and policy literature frame the study of the Directive and its effect on enhancing or constraining societal resilience by using an adaptive governance approach.
16. Karamage et al.	2016	USLE-based assessment of soil erosion by water in the Nyabarongo river catchment, Rwanda	*Environmental Research and Public Health*	Rwanda	The study indicates that Nyabarongo catchment is extremely exposed to soil erosion by water due to unmanaged intensive cropland expansion over steep slopes.
17. Schumann	2011	Flood risk assessment and management	Book	Germany	Flood probability alone is not sufficient when multiple failure modes must be considered in planning. On the other hand, operational FRM depends strongly on hydrology forecasts.
18. Ferreira et al.	2022	Nature-based solutions for flood mitigation and resilience in urban areas	Handbook of Environmental Chemistry	Urban areas	Nature-based solutions (NbS) in urban areas favour stormwater retention, infiltration and filtration, contributing to flood risk mitigation and enhancement of water quality. Their effectiveness is influenced by design and placement aspects
19. Dung et al.	2020	The role of relative slope length in flood hazard mapping using AHP and GIS	*Geography, Environment, Sustainability*	Vietnam	Researchers mapped flood hazards in Vietnam’s Lam River Basin using AHP and GIS. Findings demonstrate that adding relative slope length to standard variables significantly improves mapping accuracy. Validation against historical floods confirms the six-factor model is more detailed and reliable for identifying high-risk zones.
20. Djalante et al.	2011	Adaptive governance and managing resilience to natural hazards	*International Journal of Disaster Risk Management*	Australia	The article describes the conceptual understanding of interlinkages of adaptive governance, resilience and disaster risk reduction.
21. Freni and Liuzzo	2019	Effectiveness of rainwater harvesting systems for flood reduction in residential urban areas	*Water*	Switzerland	Rainwater harvesting effectively reduces flood volumes and events in residential urban areas, especially during low to medium rainfall. Effectiveness is limited in severe storms due to capacity constraints, highlighting that performance is highly related to rainfall intensity.
22. Habumugisha and Mukashyaka	2023	Flood management in Rwanda: An analysis of policy implementation perspective	*African Journal of Climate Change and Resource Sustainability*	Rwanda	Based on the sources provided, Rwanda’s 2012 National Disaster Management Policy achieved partial success in its objectives, particularly coordination. However, flood damage showed an upward trend (2016–2020), resulting in marginal overall success. Key factors hindering full achievement included overly optimistic expectations and inadequate local collaboration.
23. Hahirwabasenga et al.	2024	Flooding in Sebeya catchment, Rwanda: A review of causes, impacts, and management	*Internal Journal of Disaster Risk Reduction*	Rwanda	Flooding is a serious threat in Rwanda’s Sebeya catchment due to steep topography, high rainfall, climate change and human activities causing sedimentation. Impacts include loss of life and property damage. Data scarcity is a major challenge for flood management. Recommendations include increased monitoring, data collection, research, modelling and early warnings. These findings are relevant regionally.
24. Izere et al.	2024	Suitability analysis for resettlement potential sites of flood vulnerable community in Kigali City, Rwanda.	*Earth Systems and Environment*	Rwanda	Floods seriously impact Kigali’s Nyarugenge district, necessitating resettlement. Using GIS-AHP, over 50% was found suitable for resettlement, but 23.82% (30.9 km^2^) is unsuitable, requiring relocation of 7677 settlements. Suitability is influenced by elevation, proximity to rivers/services and other factors.
25. Clemence Iradukunda	2025	Assessing community vulnerability to landslide and flood in northern Rwanda	*International Journal of Disaster Risk Reduction*	Rwanda	Researchers developed a Contextualised Vulnerability Index to assess risks in northwestern Rwanda. Findings identify living conditions, demographics and economic status as primary vulnerability drivers. Landslide-prone areas exhibit the highest risk, concentrated in the western region. Notably, landlessness reduces vulnerability by facilitating greater mobility.
26. Clemence Iradukunda	2025	Evaluating disaster risk management system (DRM)	Disaster event journal	Rwanda	Rwanda’s DRM system has established frameworks but faces critical implementation gaps. The May 2023 disaster resulted in 136 deaths and widespread infrastructure destruction. Findings highlight inadequate early warnings and poor coordination. Key recommendations include enhancing community-based alerts and allocating dedicated funding.
27. Lafevor and Ramos-Scharrón	2021	Effects of hillslope trenching on surface water infiltration in subalpine forested catchments	*Hydrology*	Mexico	Infiltration trenches provided only a 1.2% increase in total infiltration over a 2-year period compared to a baseline scenario. Runoff was infrequent and naturally infiltrated downslope in baseline conditions, making trenches ineffective for runoff harvesting. The small benefit likely results from reduced interception loss in trenches.
28. Macharia et al.	2023	Streamflow and flood prediction in Rwanda using machine learning and remote sensing in support of rural first-mile transport connectivity.	*Frontiers in Climate*	Rwanda	Machine learning models such as the Gradient Boosting Machine model effectively predicted streamflow in Rwanda’s Nyabarongo catchment, outperforming a process-based model. Findings show streamflow and flood increases linked to extreme rainfall and land use change. Associations exist between weather/floods and rural trail bridge use.
29. Mwenje and Kumar	2024	Challenges for mainstreaming climate adaptation in Africa cities. A case study of Kigali, Rwanda.	*Landscape and Urban Planning*	Rwanda	A study found Kigali’s urban plans poorly mainstream climate adaptation, scoring low on awareness, analysis and action. Key challenges include a lack of awareness, data, expertise, funding and coordination. This mirrors wider issues in African cities.
30. Uwacu et al.	2021	Using radical terraces for erosion control and water quality improvement in Rwanda: A case study in Sebeya catchment	*Environmental Development*	Rwanda	Radical terraces in Rwanda’s Sebeya catchment effectively control soil erosion and improve surface water quality by reducing sediment and nutrient runoff. Although some sites exceed WHO standards, terraced areas demonstrate better indicators than non-terraced regions, particularly when coupled with vegetation cover and riser protection.
31. Nduwayezu et al.	2021	Urban growth and land use/land cover changes in the post-genocide period	*Environment and Urbanization*	Rwanda	Based on the sources, Kigali experienced rapid post-genocide urban growth, quadrupling its population. Built-up areas expanded significantly (10.24% annual rate), decreasing open land. Key drivers include rural-urban migration, socio-economic factors, and topography, resulting in horizontal sprawl.
32. Neachell	2014	Book review-Environmental flows: Saving rivers in the third millennium.	*River Research and Applications*	Turkey	The book synthesises research on environmental flows, which are controlled releases from dams advocated to restore rivers and approximate natural flow regimes. It details dam impacts and explores methods and policies for implementing flows addressing the difficult question of how much water a river needs. It is considered an important resource for scientists and policymakers.
33. Ntabakirabose et al.	2024	The impact of forest products demand on deforestation in Rwanda. A case study of Burera, Gakenke, Gicumbi, Musanze, and Rulindo Districts.	*International Journal of Agricultural and Applied Sciences*	Rwanda	The study in Rwanda links forest product demand to household factors. Deforestation is significantly driven by agriculture, urbanisation, fires and overpopulation. It causes climate change, habitat loss and decline in quality of life.
34. Ntwali et al.	2016	The impacts of topography on spatial and temporal rainfall distribution over Rwanda based on WRF model	*Atmospheric and Climate Sciences*	Rwanda	A Weather Research and Forecasting model study in Rwanda shows topography significantly impacts rainfall distribution. Reducing elevation decreased March – May rainfall, but increased it in September – December, by altering moisture transport. Mountains act as barriers promoting orographic lifting, influencing seasonal patterns differently. Model accuracy varies by region and season.
35. Perez-Guzman et al.	2023	Sustainability implications of Rwanda’s vision 2050 long-term development strategy	*Sustainability Science*	Rwanda	Modelling Rwanda’s Vision 2050 shows agricultural productivity and diet shifts can reduce cropland, increase natural land and cut agricultural greenhouse gas emissions, aiding biodiversity, However, assumed dietary changes lower per capita calorie intake versus current trends, posing a food security trade-off needing tailored diets.
36. Rutebuka et al.	2021	Effectiveness of terracing techniques for controlling soil erosion by water in Rwanda	*Journal of Environmental Management*	Rwanda	A study in Rwanda found bench terraces significantly reduced runoff and soil loss at both sites. Progressive terrace effectiveness varied; poor construction and maintenance led to higher runoff and less soil loss control at one site. Proper establishment and maintenance are crucial.
37. Sujono	2010	Flood reduction function of paddy rice fields under different water saving irrigation techniques	*Scientific Research*	Global	Paddy fields effectively reduce floods by storing rainfall, retaining over 40%. Effectiveness depends on irrigation technique. Semi-dry cultivation is the most effective, retaining 55.7% of rainfall without yield loss. Alternate Wetting and Drying and traditional methods are less effective.
38. Uwizewe et al.	2024	Investigation of the historical trends and variability of rainfall patterns during the March – May season in Rwanda	*Atmosphere*	Rwanda	The study found a general downtrend in Rwanda’s crucial March-May rainfall (1983–2021), with a significant decrease in the southwest. Rainfall shows marked spatial variability (southwest-northwest gradient) and is influenced by Indian Ocean and South Atlantic SSTs and atmospheric circulation. CHIRPS data accurately reflects these patterns.
39. Zhang et al.	2022	Effects of check dam on sediment load under vegetation restoration in the Hekou-Longmen Region of the Yellow River	*Frontiers in Environmental Science*	China	The Yellow River’s Hekou-Longmen region saw a significant sediment load decrease, primarily (93.21%) due to human activities. Check dams are more effective in basins with low vegetation coverage. Vegetation restoration alone has certain upper limits, so combining both yields greater benefits.
40. Niyomugabo et al.	2022	View of the role of rain forecasting in flood risk reduction. Case study of Kigali City	*International Journal of Climate Research*	Rwanda	The study found that precipitation significantly influences flood risks in Kigali, particularly in the Nyarugenge district, which faced the highest impacts. Results show strong positive correlations between rainfall and deaths, injuries, and infrastructure losses. Findings highlight using rain forecasting to improve policy, land use and community resilience.
41. Nzabonantuma et al.	2025	Flood susceptibility mapping in the Nyabarongo Catchment, Rwanda, based on data analysis and modelling	*Geomatics, Natural Hazards and Risk*	Rwanda	The Random Forest model was the most accurate (AUC 0.968) for mapping flood susceptibility in Rwanda’s Nyabarongo catchment. Distance to river, TWI, and slope were the most influential predictors. Significantly, 15.6% of the region faces high to very high flood risk

FRM, flood risk management; WHO, World Health Organization; NDVI, Normalized Difference Vegetation Index; USLE, Universal Soil Loss Equation; AHP, Analytic Hierarchy Process; GIS, Geographic Information System; CHIRPS, Climate Hazards Group InfraRed Precipitation with Station data; AUC, Area Under the Curve; TWI, Topographic Wetness Index.

**TABLE 3-A1:** Publications from stakeholders.

Authors	Year	Title	Type	Study area	Main findings
1. MINEMA	2023	Disaster effects situation from 03 to 22 May 2023	Report	Rwanda	The report presents the impacts that occurred between 03 and 22 May 2023 across Rwanda. Floods and landslides caused by prolonged and intense rainfall were the main sources of losses.
2. REMA	2020	Completion of technical assistance in environment and natural resources management	Catchment management plan	Upper Akagera catchment, Rwanda	The development of a catchment management plan for the Nile Akagera Upper Catchment
3. NISR	2023	5th Population and housing census, Rwanda, 2022	Census report	Rwanda	It published the population count, evolution, distribution and structure in Rwanda in 2022.
4. REMA	2021	Rwanda state of environment and outlook	Report	Rwanda	The report provides the status of the environment in Rwanda, and guidelines and technical assistance to sustain ecosystem services.
5. RWB and IUCN	2022	The state of erosion control in Rwanda	Report	Rwanda	Soil erosion risks 45% of Rwanda’s land, but only 26% is currently protected. Seasonal agricultural productivity losses reach 37.9 billion RWf, while annual topsoil loss values hit 810bn RWf. Restoration requires 514bn RWf over a decade, prioritising contour terraces, afforestation and stormwater management.
6. Ministry of Emergency (MINEMA)	2024	Disaster effects situation report	Report	Rwanda	It reports disaster impacts that occurred throughout 2024, with flood- and rainstorm-related damage accounting for most losses.
7. REMA	2018	Rwanda environment statistics compendium, 2018	Report	Rwanda	The report is a compilation of existing data from governmental ministries, authorities, agencies, non-governmental organisations and data produced by the National Institute of Statistics of Rwanda (NISR).
8. International Monetary Fund	2023	Resilience and sustainability facility	Report	Rwanda	Rwanda remains highly vulnerable to climate disasters, evidenced by devastating 2023 floods. Despite innovative finance and carbon frameworks, a $11bn funding gap and high debt persist. International support and private investment remain vital for building resilience and transitioning to a low-carbon economy.
9. The World Bank	2024	Rwanda disaster risk management development policy	Report	Rwanda	The US$142 million operation aims to strengthen Rwanda’s capacity to manage climate, disaster and health risks. It focuses on improving institutional coordination, building resilient infrastructure and enhancing natural resource management. These reforms are targeted to protect economic growth and reduce vulnerability for poor households.
10. The World Bank	2017	Reshaping urbanisation in Rwanda	Report	Rwanda	Rwanda’s secondary cities grow slower than Kigali, with urban sprawl outpacing densification. The Rubavu-Musanze corridor holds the most economic potential outside the capital. Major constraints include infrastructure deficits, housing unaffordability and limited fiscal autonomy. Sustainable growth requires prioritising high-potential areas and strengthening urban governance.
11. MINEMA	2023	National disaster risk reduction and management policy	Policy	Rwanda	It revised the 2011 disaster management (DM) policy and transferred all disaster management related responsibilities to MINEMA and its partners. The revised policy outlines roles and responsibilities for dealing with disasters.
12. MINEMA	2024	Disaster risk mitigation plan for CENR sector	Report	Rwanda	Rwanda’s plan mitigates climate-induced floods, landslides and droughts through integrated land, water and forest management. It prioritises reforestation, terracing and resilient infrastructure while addressing financial gaps. Community-led Eco-DRR, early warning systems and gender mainstreaming are essential for ensuring long-term national resilience.
13. Global Water Leadership Rwanda Programme	2024	Strengthening water	Report	Rwanda	Rwanda faces water scarcity despite high rainfall. Key findings highlight bottlenecks in technical capacity, flood management, community awareness and siltation. The Rwf 19.8bn strategy prioritises soil conservation, capacity building and inclusive governance to enhance climate resilience and ensure sustainable growth.
14. National Council for Research in Science and Technology	2023	NCST quarterly Newsletter	Newsletter	Rwanda	Rwanda is accelerating its knowledge-based economy by integrating 4IR technologies and establishing Grand Challenges Rwanda to solve social problems. Key partnerships with Israel focus on ecosystem analysis, while national efforts prioritise gender parity in STEM through the International Day of Women and Girls in Science.
15. Government of Rwanda (GoR)	2024	Five years government programme	Report	Rwanda	Rwanda’s NST2 (2024–2029) targets 9.3% GDP growth and 1.25 million jobs. It prioritises doubling private investment, boosting exports and achieving universal electricity. Social targets include reducing stunting to 15%, while governance focuses on digital transformation and 85% citizen satisfaction.
16. Ministry of Environment (MoE)	2022	Green growth and climate resilience: National strategy for climate change and low carbon development	Report	Rwanda	Rwanda’s strategy targets a carbon-neutral, high-income economy by 2050. It focuses on green industrialisation, smart urbanisation and sustainable land management. Implementation requires 700 million in public spending, aiming for 60% renewable energy and 70% urbanisation to secure national resilience.
17. Rwanda Water Resources Board (RWB)	2023	Baseline biophysical and socio-economic assessment for landscape restoration and integrated water resources management in Sebeya catchment	Report	Rwanda	The sources report soil erosion threatens 45% of Rwanda’s land, causing annual productivity and topsoil losses worth 810bn RWf. With only 26% currently protected, restoration requires 514bn RWf through community-led IWRM and VLUAP strategies. Catchments face future water stress, necessitating optimised management to ensure national resilience.
18. MoE	2022	Official Gazette no Special of 30/07/2021: Ministerial order governing the water resources management committee at catchment level and the technical committee on dams.	Statute	Rwanda	Rwanda’s 2018–2024 strategy optimises natural capital to secure high-income status by 2050. It scales green finance via FONERWA and targets 30% forest cover while doubling agroforestry land. By professionalising mining and adopting Natural Capital Accounting, the plan mitigates climate risks while aiming for 3.6% GDP.
19. RWB	2021	Rwanda Water Resources Board strategic plan (2021–2030)	Statute	Rwanda	The Rwanda Water Resources Board’s 2021–2030 Strategic Plan focuses on securing water availability and mitigating climate risks. Addressing critical soil degradation affecting 55% of the country, the framework establishes five core objectives: strengthening governance, controlling erosion, enhancing water storage, building disaster resilience and ensuring institutional sustainability.

REMA, Rwanda Environment Management Authority; DRR, disaster risk reduction; 4IR, Fourth Industrial Revolution; NST, National Strategy for Transformation; NCST, National Council of Science and Technology; GDP, gross domestic product; IUCN, International Union for Conservation of Nature; RWF, Rwandan Francs; CNR, Curve Number Ratio; STEM, Science, Technology, Engineering and Mathematics; IWRM, Integrated Water Resources Management; VLUAP, Village Land Use Action Plan; FONERWA, Rwanda Green Fund.
